# The incidence and clinical features of PEGylated filgrastim-induced acute aortitis in patients with breast cancer

**DOI:** 10.1038/s41598-020-75620-6

**Published:** 2020-10-29

**Authors:** Sang Yoon Lee, Eun Kyoung Kim, Ji-Yeon Kim, Taek-kyu Park, Seung-Hyuk Choi, Young-Hyuck Im, Min Yeong Kim, Yeon Hee Park, Duk-Kyung Kim

**Affiliations:** 1 Division of Cardiology, Department of Medicine, Heart Vascular Stroke Institute, Samsung Medical Center, Sungkyunkwan University School of Medicine, #81 Irwon-ro, Gangnam-gu, Seoul, 06351 Republic of Korea; 2 Division of Hematology-Oncology, Department of Medicine, Samsung Medical Center, Sungkyunkwan University School of Medicine, Seoul, Republic of Korea; 3 Department of Radiology, Samsung Medical Center, Sungkyunkwan University School of Medicine, Seoul, Republic of Korea

**Keywords:** Cardiology, Medical research, Oncology

## Abstract

Although PEGylated filgrastim-induced aortitis is very rare and unknown clinically, some cases were reported and increasing, especially in breast cancer patients. The present study investigated the prevalence, clinical features and treatment of aortitis induced by PEGylated filgrastim in patients with breast cancer. A total of 2068 consecutive patients who underwent neoadjuvant/adjuvant chemotherapy with PEGylated filgrastim for breast cancer were enrolled. From the medical record, clinical, laboratory, medication, and imaging evaluation findings were collected. PEGylated filgrastim-induced aortitis was established in 0.3% of the study population. Common clinical presentations included extremely high fever and chest/back pain with high levels of inflammatory markers without any signs of infection. Contrast-enhanced computed tomography scans revealed typical enhancing wall thickening and periaortic soft tissue infiltration at various levels of aorta. All patients improved rapidly after treatment with modest doses of prednisolone (0.5 mg/kg/day) without any complications. Clinicians should be aware of aortitis as a possible complication of granulocyte-colony stimulating factor therapy, especially PEGylated filgrastim, given the frequent misdiagnoses in neutropenic patients undergoing chemotherapy.

## Introduction

Aortitis is a rare clinical manifestation caused by multiple etiologies including infection or autoimmune disease^1,2^. While several drugs such as ergot alkaloids, dopaminergic drugs or methysergide have been known to be the primary cause of chronic aortitis, drugs that cause acute aortitis are very rarely reported^2^. Recombinant human granulocyte-colony stimulating factors (G-CSF) are widely used for primary or secondary prevention of chemotherapy-induced neutropenia in patients treated with anticancer agents. The use of G-CSF is generally considered safe with relatively mild side effects such as bone pain^3^. However, critical cardiovascular adverse events including arterial thrombosis or aortitis are rarely reported^4–6^.

In clinical field, there are several kinds of G-CSF agents. Filgrastim and lenograstim, which is a short-acting agent of G-CSF, have very similar biological structure and activity compared to endogenous human G-CSF; PEGylated filgrastim is a long-acting agent of G-CSF^7^. According to cases reported to date, several types of G-CSF (filgrastim, lenograstim, lipegfilgrastim and PEGylated filgrastim) can cause aortitis in patients with various types of malignancies^6,8–20^. The exact incidence of G-CSF related aortitis is unknown; however, it was more frequently detected in patients with breast cancer, ovarian cancer or lymphoma^20^. In particular, PEGylated filgrastim, usually used for primary prophylaxis of neutropenia in breast cancer patients, is known to be associated with the highest incidence of aortitis among G-CSF agents.

Despite the small but increasing number of cases reported for the past 10 years, the incidence, clinical characteristics and management of G-CSF-related aortitis are still unclear. In the absence of large-scale data other than those derived from case reports or adverse drug reports, we sought to investigate the prevalence, clinical features and treatment of G-CSF-induced acute aortitis in patients treated with adjuvant or neoadjuvant chemotherapy and PEGylated filgrastim for breast cancer.

## Methods

### Study population and design

We retrospectively reviewed the medical records of breast cancer patients who received PEGylated filgrastim during adjuvant or neoadjuvant chemotherapy from March 2015 to February 2020. In study population, two kinds of PEGylated filgrastim were used, which were pegfilgrastim (Neulasta-prefilled syringe 6 mg, Kyowa Hakko Kirin Co. Ltd) and tripegfilgrastim (Dulastin-prefilled syringe 6 mg, Dong-A ST Co. Ltd). The following baseline clinical data for PEGylated filgrastim-induced aortitis patients were collected: age, gender, comorbidity, chemotherapy regimen, G-CSF type, frequency of pegfilgrastim and other G-CSF administration before aortitis. Symptoms, time to onset, and extent of disease confirmed by computed tomography (CT) scans were retrieved as clinical manifestations. Laboratory tests including white blood cell counts, erythrocyte sedimentation rate (ESR), high-sensitivity C-reactive protein (CRP), procalcitonin, immunoglobulin, blood culture, and serologic markers of atypical pathogens and rheumatologic diseases were also conducted. This study was conducted in accordance with the Declaration of Helsinki and approved by the local institutional review board of Samsung Medical Center. (IRB File No. 2019-10-095-001) All of the methods were carried out in accordance with the approved guidelines and relevant regulations. Informed consent was waived for this retrospective study.

### Diagnosis of acute aortitis

Based on clinical presentation including fever, myalgia, or chest/back pain without other infectious causes, CT scan of the entire aorta was performed with contrast enhancement. On CT scan, acute aortitis was defined by aortic inflammation as well as newly-developed enhancing aortic wall thickening with peri-aortic infiltration. To exclude other causes of aortitis, blood culture and serologic tests for atypical pathogens, autoimmune diseases or connective tissue diseases were also reviewed. The history of other treatment regimens, especially chemotherapy, was reviewed to exclude other drug-induced aortitis.

### Statistical analysis

Baseline characteristics were described as numbers and percentages for all breast cancer patients treated with neoadjuvant or adjuvant chemotherapy. Continuous variables were presented as medians, standard deviation. Categorical variables were presented as percentages. All statistical analyses were performed using SPSS Statistics 23.0 (SPSS, Chicago, IL, USA).

## Results

### Baseline characteristics

A total of 2068 patients who underwent neoadjuvant/adjuvant chemotherapy with PEGylated filgrastim for breast cancer were consecutively enrolled in this study. Among study population, 1438 patients (69.5%) received pegfilgrastim, 567 patients (27.4%) received tripegfilgrastim, which is a biosimilar of pegfilgrastim, and 63 patients (3.0%) had the history of both pegfilgrastim and tripegfilgrastim administration. Mean age of study populations was 49.9 years and 2065 patients (99.9%) were female. In the study population, 470 patients (22.7%) had a history of treatment with filgrastim due to febrile neutropenia. No lenograstim treatment history was found in the study population. These results are shown in Table 1.

**Table 1:** Baseline characteristics of total study population.

	Breast cancer patients with PEGylated filgrastim (n = 2068)
Age, years	49.9 ± 11.1
**Sex**	
Female	2065 (99.9%)
Male	3 (0.1%)
**Chemotherapy indication**	
Neoadjuvant	995 (48.1%)
Adjuvant	1073 (51.9%)
**Chemotherapy regimen**	
Doxorubicin + cyclophosphamide	243 (11.8%)
Docetaxel + cyclophosphamide	383 (18.5%)
Docetaxel + doxorubicin + cyclophosphamide	724 (35.0%)
Docetaxel + carboplatin	369 (17.8%)
Others	349 (16.9%)
**G-CSF with chemotherapy**	
PEGylated filgrastim	2068 (100%)
Pegfilgrastim	1438 (69.5%)
Tripegfilgrastim	567 (27.4%)
Pegfilgrastim and tripegfilgrastim	63 (3.0%)
**G-CSF for febrile neuropenia**	
Filgrastim	470 (22.7%)
Lenograstim	0 (0%)

### Clinical manifestations

Among 2068 consecutive patients who were treated with PEGylated filgrastim once or more, six patients (0.3%) developed acute non-infectious aortitis. The median age of patients diagnosed with acute aortitis was 51.7 years. Two patients (Cases #1 and #2) developed aortitis during neoadjuvant chemotherapy, which was consist of docetaxel and carboplatin. And four patients (Cases #3–6) had aortitis during adjuvant chemotherapy. In past medical history, there was no specific features related with giant cell arteritis or Takayasu arteritis. Acute symptoms related to aortitis including extremely high fever, chest/back pain and severe myalgia occurred 13.4 ± 7.6 days after administration of pegfilgrastim. While three patients (Cases #3, #4 and #6) developed aortitis when the the first dose of PEGylated filgrastim was conducted, other patients (Cases #1, #2 and #5) developed aortitis after more than three times of PEGylated filgrastim injection. Except one patient (Case #1) who had filgrastim instead of PEGylated filgrastim after experience of aortitis, G-CSF was no longer administered for other patients. In that patient who had filgrastim injection, instead of pegfilgrastim afterwards, aortitis was recurred with symptoms similar to the first episode. And inflammatory change of the aorta was observed in the same location with the first episode.

### Laboratory and CT findings

In all patients, high fever (more than 39 °C) and high levels of CRP (24.1 ± 7.7 mg/dL) were identified without any infection-related signs. To evaluate infectious aortitis, blood and urine culture were done and assays for *Bartonella* Ab, *Brucella* Ab, and Q fever antibody were conducted to distinguish atypical pathogens. Diagnostic tests for fluorescent antinuclear antibody, antineutrophil cytoplasmic antibody, rheumatoid factor and serum levels of immunoglobulin G4 (IgG4) subclass were also conducted for rheumatologic diseases, such as polyangiitis with granulomatosis or IgG4-related diseases. All serologic tests and cultures for infection or rheumatologic disease were negative in all cases.

**Figure 1:**
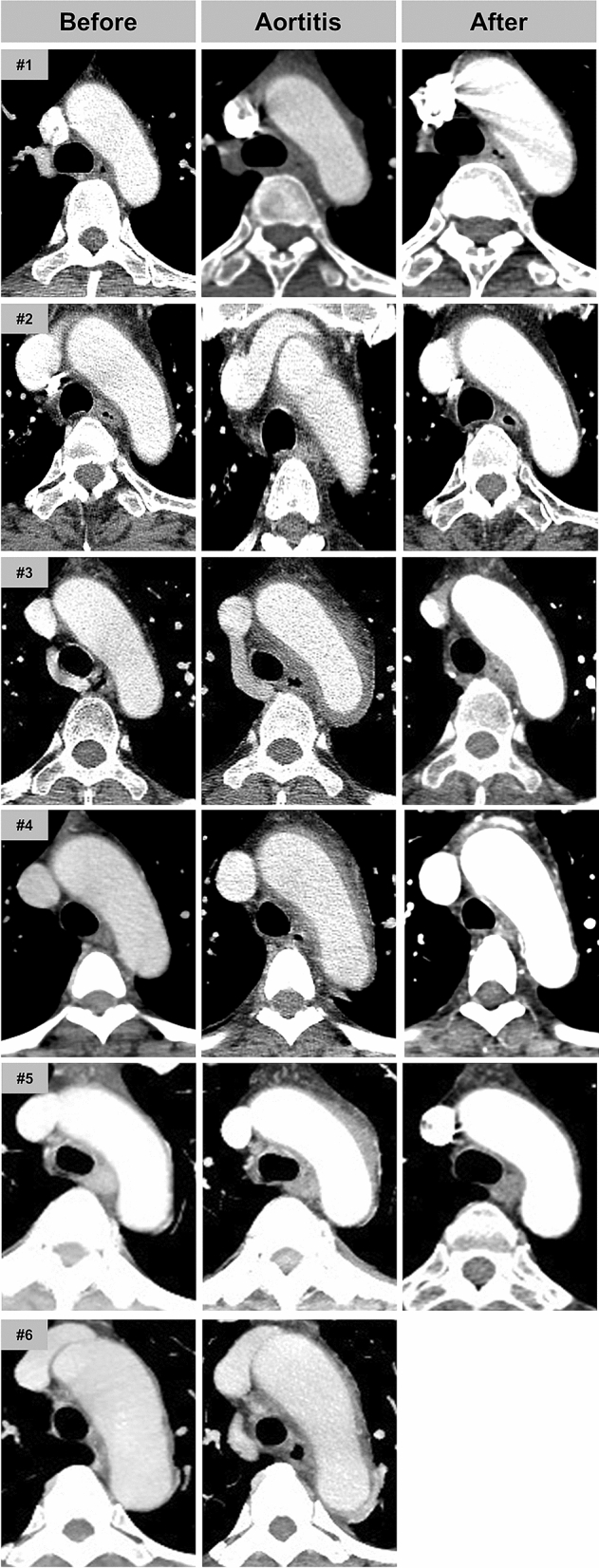
Serial CT images of aortic arch before, during and after PEGylated filgrastim-induced aortitis. Irregular wall thickening with periaortic infiltration is commonly observed in the aortic arch (Case #1–#6). After steroid treament, inflammatory changes of aortic wall were significantly improved.

**Figure 2:**
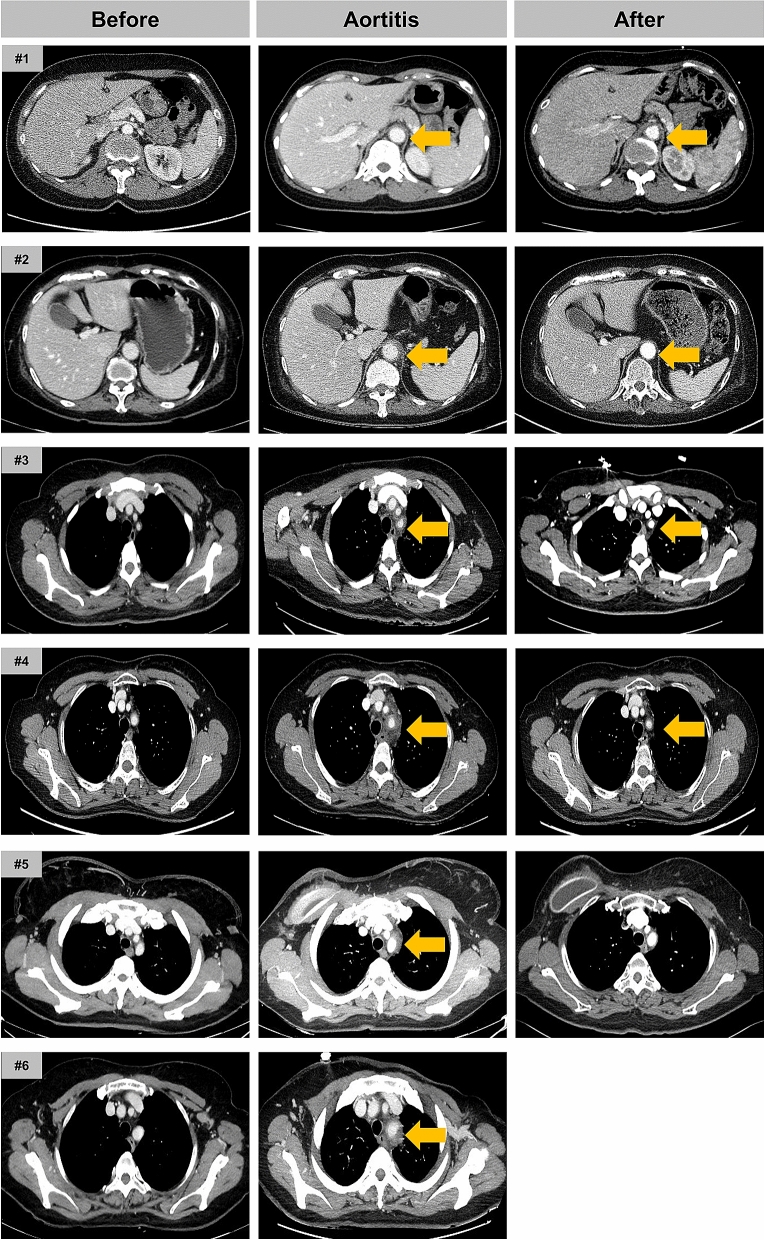
Various level of the aorta involvement in PEGylated filgrastim-induced aortitis. Case #1 and Case#2: abdominal aorta was involved. Case #3–#6: branching arteries from the aortic arch were involved.

The contrast-enhanced CT revealed enhanced segmental wall thickening of aorta with varying degrees of peri-aortic infiltration in all patients. These findings were consistently seen in the aortic arch (Fig. 1) and occasionally extended to branch arteries (Case #3–6, Fig. 2). Peri-aortic infiltration was extended to thoracoabdominal junction of the aorta in two patients (Case #1 and #2). One patient (Case #3) underwent positron-emission tomography-CT (PET-CT), which showed an increased FDG uptake (SUVmax = 3.2) along the wall of aortic arch confirming acute vasculitis (see Supplementary Fig. S1 online).

### Treatment of acute aortitis

**Figure 3:**
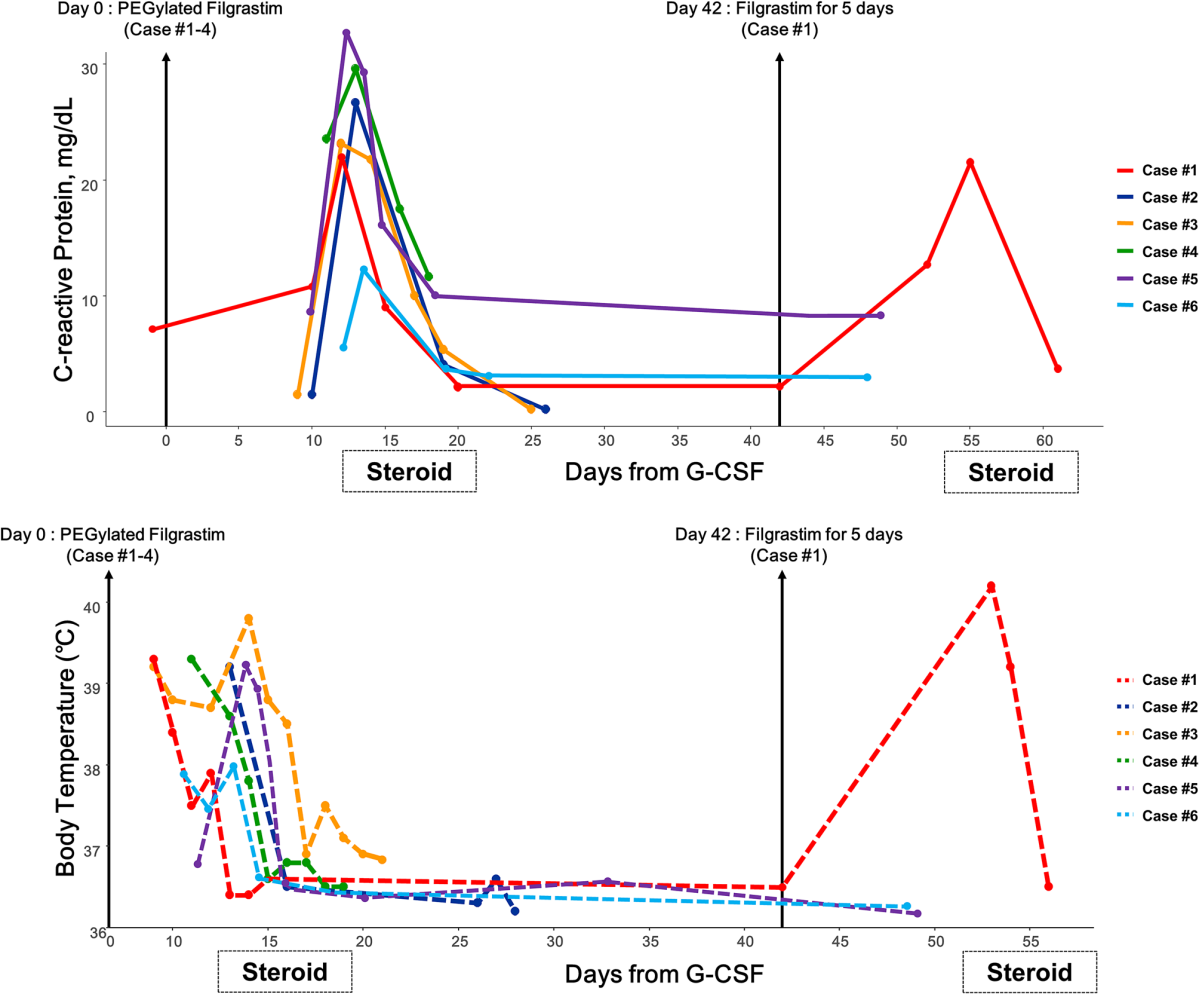
Clinical course of PEGylated filgrastim-induced aortitis. C-reactive protein (CRP) and body temperature during the clinical course of PEGylated filgrastim-induced aortitis. CRP level and fever rapidly improved after steroid administration in cases #1, #4, #5 and #6. In cases #2 and #3, CRP and fever already improved at the time of steroid administration.

Treatment with pegfilgrastim was discontinued, and prednisolone 0.5 mg/kg/day was initiated. Initial symtoms and inflammatory markers were rapidly resolved within one week. After confirming the negative test results of infection and rheumatologic diseases, patients were discharged. Steroid treatment was tappered over 1–2 months. In one patient (Case #1) who recurred aortitis after injection of filgrastim, warranted additional steroid therapy for 2 weeks. No further G-CSF treatment was administered in all patients during the remainder of chemotherapy. No significant post-aortitis complications or delay of scheduled chemotherapy were observed. The follow-up CT scans after more than 1 month from onset revealed a remarkable improvement in wall thickening and infiltration. Detailed clinical features and the result of treatment are depicted in Table 2 and Fig. 3.

**Table 2:** PEGylated filgrastim-induced aortitis among breast cancer patients treated with neoadjuvant or adjuvant chemotherapy.

	Case 1	Case 2	Case 3	Case 4	Case 5	Case 6
**Baseline characteristics**						
Age	45 years	66 years	49 years	50 years	59 years	53 years
Gender	Female	Female	Female	Female	Female	Female
Race	Asian	Asian	Asian	Asian	Asian	Asian
Comorbidity	None	Hypertension Hypothyroidism	Hypertension	None	None	None
Chemotherapy indication	Neoadjuvant	Neoadjuvant	Adjuvant	Adjuvant	Adjuvant	Adjuvant
Chemotherapy regimen	Docetaxel Carboplatin	Docetaxel Carboplatin	Docetaxel Cyclophosphamide	Docetaxel Cyclophosphamide	Docetaxel Carboplatin Trastuzumab Hertuzumab	Docetaxel Cyclophosphamide
G-CSF type at the onset of aortitis	Pegfilgrastim Filgrastim(Recurrence)	Pegfilgrastim	Pegfilgrastim	Pegfilgrastim	Pegfilgrastim	Pegfilgrastim
Frequency of pegfilgrastim before aortitis	5	3	1	1	1	4
**Clinical manifestations**						
Symptoms	Fever Myalgia Chilling Epigastric discomfort	Fever Chilling Myalgia Nausea	Fever Chest discomfort Dyspepsia Myalgia	Fever Chilling Myalgia	Fever Myalgia Chilling	Fever Chilling Myalgia Headache
Physical examination	Abdominal tenderness	Abdominal tenderness	Unremarkable	Unremarkable	Unremarkable	Unremarkable
Time to onset	(1st) 12 days (2nd) 10 days	13 days	15 days	12 days	17 days	14 days
Extent of disease	Aortic arch Abdominal aorta	Aortic arch Left CCA Right innominate artery Thoracic aorta Abdominal aorta	Aortic arch Left CCA	Aortic arch Right innominate artery Left SCA Left CCA	Aortic arch Left CCA Right innominate artery Thoracic aorta Abdominal aorta	Aortic arch Left CCA
**Laboratory findings**						
WBC count, × 10^3^/μL	7.46 (Neutrophil-dominant)	19.76 (Neutrophil-dominant)	12.33 (Neutrophil-dominant)	38.29 (Neutrophil-dominant)	16.91 (Neutrophil-dominant)	11.25 (Neutrophil-dominant)
ESR, mm/h	53	118	119	69	120	64
CRP, mg/dL	23.11	26.63	21.76	29.58	32.86	10.85
Procalcitonin, ng/mL	0.04	0.26	0.18	0.05	0.28	0.04
Blood culture	Negative	Negative	Negative	Negative	Negative	Negative
Infection serology^a^	Negative	Negative	Negative	Negative	Negative	Negative
Rheumatologic markers	RF/FANA/ANCA (–/–/–)	RF/FANA/ANCA (–/–/–)	RF/FANA/ANCA (–/–/–)	RF/FANA/ANCA (–/–/–)	RF/FANA/ANCA (–/–/–)	No data
Immunoglobulin (IgG4)	No data	No data	Normal	Normal	Negative	No data

*CCA* common carotid artery, *SCA* subclavian artery.

^a^Infectious markers including following tests: syphilis, *Salmonella, Borrelia, Mycoplasma, Chlamydia*, *Richettsia*, Q fever, *Brunella, Bartonella, Legionella, M. tuberculosis.*

## Discussion

Drug-induced acute aortitis is extremely rare, but is a critical co-morbidity, especially in cancer patients requiring continuous chemotherapy. Here, we reported the incidence and clinical manifestations of PEGylated filgrastim-induced acute aortitis in breast cancer patients who underwent chemotherapy. Among the 2068 patients who received PEGylated filgrastim for prophylaxis of neutropenia, six patients developed acute aortitis (0.3%). Common clinical presentations included extremely high fever and chest/back pain with high levels of CRP without any signs of infection. The onset of aortitis usually occurred within 2 weeks after PEGylated filgrastim injection. The contrast-enhanced CT scans revealed aortic enhancing wall thickening and peri-aortic soft tissue infiltration at various levels of aorta, most commonly along the aortic arch. All patients rapidly improved after administration of a modest dose of prednisolone without any complications and returned to chemotherapy as scheduled. To the best of our knowledge, this is the first study to report detailed clinical features of PEGylated filgrastim-induced acute aortitis during chemotherapy in patients with breast cancer.

In breast cancer patients, dose-dense chemotherapy involving administration of chemotherapy agents more frequently than typical chemotherapy, resulted in a significant improvement of clinical outcomes^21,22^. However, this intensified chemotherapy induces additional neutropenia and infection-related complications^23^. To prevent chemotherapy-induced febrile neutropenia, G-CSF was added to chemotherapy regimen and chemotherapy with PEGylated filgrastim yielded favorable clinical outcomes in breast cancer patients^24,25^. Therefore, considering the fact that most of the breast cancer patients undergoes chemotherapy with PEGylated filgrastim, aortitis could occur more easily in breast cancer patients. However, for breast cancer, few data pertained to G-CSF-related aortitis including several case reports^6,8–15,17–19^ and two study from Adverse Drug Reporting (ADR) systems^16,20^. Data from the United States Food and Drug Administration Adverse Event Reporting System (FAERS) reported that the frequency of G-CSF-related aortitis was 0.0014%^20^. Another data based on the Japanese ADR database reported the incidence of G-CSF-induced aortitis as 0.47%^20^. Although those two results showed the association between G-CSF injection and the development of aortitis, they included the patients with malignancy and not exposed to chemotherapy with G-CSF in study population and did not report the detailed clinical courses including treatment of aortitis. Considering that chemotherapy with PEGylated filgrastim is widely used to treat breast cancer patients, one of the strengths of our study relates to the real-world incidence in consecutive patients and a comprehensive description regarding the clinical course of PEGylated filgrastim-induced aortitis in patients with breast cancer.

G-CSF stimulates proliferation and diffrentiation of neutriphil precursors, enhances chemotaxis and mobilizes hematopoietic stem cells^26^. Aortitis might be triggered via increased pro-inflammatory reaction and neutrophil-mediated damage^27^. Additionally, G-CSF-induced autoimmune reactions mediated via IL-6 between Th17 cells and CD4+ T cells is also thought to result in aortitis, aneurysm or dissection^9,10,28^. In fact, blood tests at the onset of PEGylated induced-aortitis patients showed a significant increase in G-CSF as well as neutrophil and cytokine levels^10^. Based on this immune-related vascular inflammation as one of the main mechanisms underlying the development of aortitis, all our patients were treated with short-term steroids to induce immunosuppression. While some data showed spontaneous improvement of aortitis without anti-inflammatory agents^8,11^, we thought that the recovery without anti-inflammatory agents might take longer than for the use of glucocorticoid, and delay recovery could directly affect the anticancer treatment schedule. Anticipating no further delay in chemotherapy, it may be reasonable to consider short-term glucocorticoid treatment. Further studies are warranted to determine the appropriate duration of steroid administration and indications for the restart of chemotherapy.

Early diagnosis of G-CSF-induced aortitis can be established based on high fever and chest/back pain combined with remarkable elevation in inflammatory markers, when G-CSF administration is known. Despite the high levels of CRP and ESR, these patients appear to be stable without symptoms related to infection or autoimmune disease. There have been a difference of the time from G-CSF administration to the onset of aortitis among the studies. It might vary depending on how aortitis-indicating symptoms were defined. Because mild fever and general ache are frequently observed in cancer patients after chemotherapy regardless of G-CSF administration, we defined aortitis-indicating symptoms as extremely high fever and localizing chest/back pain without other infection-related signs. Therefore, the symptom onset time was longer than the previous data^17^. Immediate chest CT or CT aortography is indicated considering the risk of disease spread to proximal aorta, such as aortic arch or ascending thoracic aorta. Of course, even if G-CSF induced aortitis is suspected, the tests for infection and rheumatic disease are required at the same time. Procalcitonin, which is associated with the extent and severity of bacterial infection^29^, was within normal range despite the highly elevated ESR/CRP. This finding may facilitate to exclude the diagnosis of other suspected causes of fever and inflammation.

A comprehensive review of past medications for the patients with aortitis are also important. Few case reports showed the aortitis caused by gemcitabine or bevacizumab^30–32^. Similary with the preivous data^8,11–13,17–19^, docetaxel was concorrently used in our aortitis patients. However, aortitis did not recur during the remainder of docetaxel chemotherapy without G-CSF after aortitis. Therefore, we thought docetaxel was not the primary cause of aortitis. Some data have suggested there might be a potential interaction between taxanes and G-CSF, and their synergistic effect might promote vasculitis^17,33^.

Compared with the other cases discontinuing G-CSF treatment, Case #1 was exposed to filgrastim instead of pegfilgrastim after the resolution of the initial aortitis-related symptoms. Except our data (Case #1), no report of recurrent aortitis after administration of another type of G-CSF has been reported. Clinicians should be cautious when re-administration of G-CSF, even if it is a different type of G-CSF with the prior agent, considering the possibility of cross-reaction between drugs. Notably, in the present study, all cases of aortitis were induced by pegfilgrastim, which is an original PEGylated filgrastim made by Kyowa Hakko Kirin Co. Ltd. Among patients who received tripegfilgrastim, which is a biosimilar of pegfilgrastim, no aortitis occurred. According to previous studies including data from Japan ADR database^6,8–20^, the biosimilars of G-CSF did not cause aortitis. Taken together, although the mechanism is unknown, biosimilars of G-CSF can be the alternatives when G-CSF administration is inevitable for patients.

According to the previous studies to date, the incidence of G-CSF induced aortitis is higher in East Asian. Pegfilgrastim is world widely used G-CSF, so the difference in the incidence of G-CSF induced aortitis might not be due to the difference in the rate of drug use. One of the possible explanation is that a certain genetic predisposition might be related to the increased incidence of G-CSF induced aortitis in Asian. Th17 cell pathway, which is considered as one of the pathogenesis of G-CSF induced aortitis, is also known to contribute to the systemic and vascular manifestations of Takayasu’s arteritis that is common in East Asia^10,34^. Genetic susceptibility to large vessel inflammatory change might have caused differences in the frequency of drug-related vasculitis between races. Underdiagnosis should be considered as another explanation for relatively low incidence of G-CSF induced aortitis in Western. In terms of G-CSF induced aortitis, it is difficult to diagnose without clinical suspicion and early imaging evaluation. Future large scale studies are warranted to identify the exact incidence of G-CSF induced aortitis and bring awareness to physicians.

The present study had several limitations. First, this was a single center retrospective study. Second, the number of patients was relatively small; however, all patients were enrolled consecutively and our study focused on discrete manifestations of aortitis. Lastly, among patients who presented with fever, chest/back pain and elevated inflammatory markers, patients were diagnosed with pegfilgrastim-related aortitis only when the newly developed aortic wall thickening was detected in imaging tests, the temporal relationship was evident following pegfilgrastim injection, and the exclusion of infection and autoimmune disease was established. Because of the strict diagnositc criteria for aortitis, the exact incidence might be underestimated.

In conclusion, among breast cancer patients who were treated with adjuvant/neoadjuvant chemotherapy including PEGylated filgrastim, the incidence of PEGylated filgrastim-induced acute aortitis was 0.3%. Although not established as a treatment of choice, short-term steroid administration and avoidance of all G-CSF types can be effective in these patients. In view of the remarkable systemic symptoms associated with aortitis, which can be misdiagnosed as infection in neutropenic patients undergoing chemotherapy, clinicians should be aware of aortitis as a possible complication of G-CSF therapy, especially in breast cancer patients treated with PEGylated filgrastim.

## Supplementary information

Supplementary Figure.
