# Supplementary material for: The incidence and clinical features of PEGylated filgrastim-induced acute aortitis in patients with breast cancer
Source: Sci Rep. 2020 Oct 29;10:18647. doi: 10.1038/s41598-020-75620-6 (PMC7596224; doi:10.1038/s41598-020-75620-6)

**The Incidence and Clinical Features of PEGylated Filgrastim-induced Acute Aortitis in Patients with Breast Cancer**

Sang Yoon Lee^1^, Eun Kyoung Kim^1*^, Ji-Yeon Kim^2^, Taek-kyu Park^1^, Seung-Hyuk Choi^1^, Young-Hyuck Im^2^, Min Yeong Kim^3^, Yeon Hee Park^2^, Duk-Kyung Kim^1^

^1^Division of Cardiology, Department of Medicine, Heart Vascular Stroke Institute, Samsung Medical Center, Sungkyunkwan University School of Medicine, Seoul, Republic of Korea

^2^Division of Hematology-Oncology, Department of Medicine, Samsung Medical Center, Sungkyunkwan University School of Medicine, Seoul, Republic of Korea

^3^Department of Radiology, Samsung Medical Center, Sungkyunkwan University School of Medicine Seoul, Republic of Korea

^*^**Address for Correspondence**

Eun Kyoung Kim, MD., PhD

Division of Cardiology, Department of Medicine, Heart Vascular Stroke Institute,

Samsung Medical Center, Sungkyunkwan University School of Medicine

#81 Irwon-ro, Gangnam-gu, Seoul, 06351, Korea

Tel: 82-2-3410-3419;FAX: 82-2-3410-3849;E-mail: [ekbobi.kim@samsung.com](mailto:ekbobi.kim@samsung.com)

**Supplementary Figure S1 : Positron-emission Tomography-CT (PET-CT) of Case #3**

PET-CT scans show linear FDG uptake along the thickened wall of aortic arch.


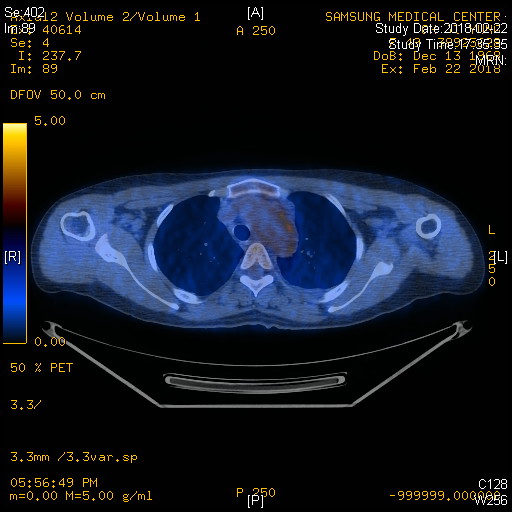

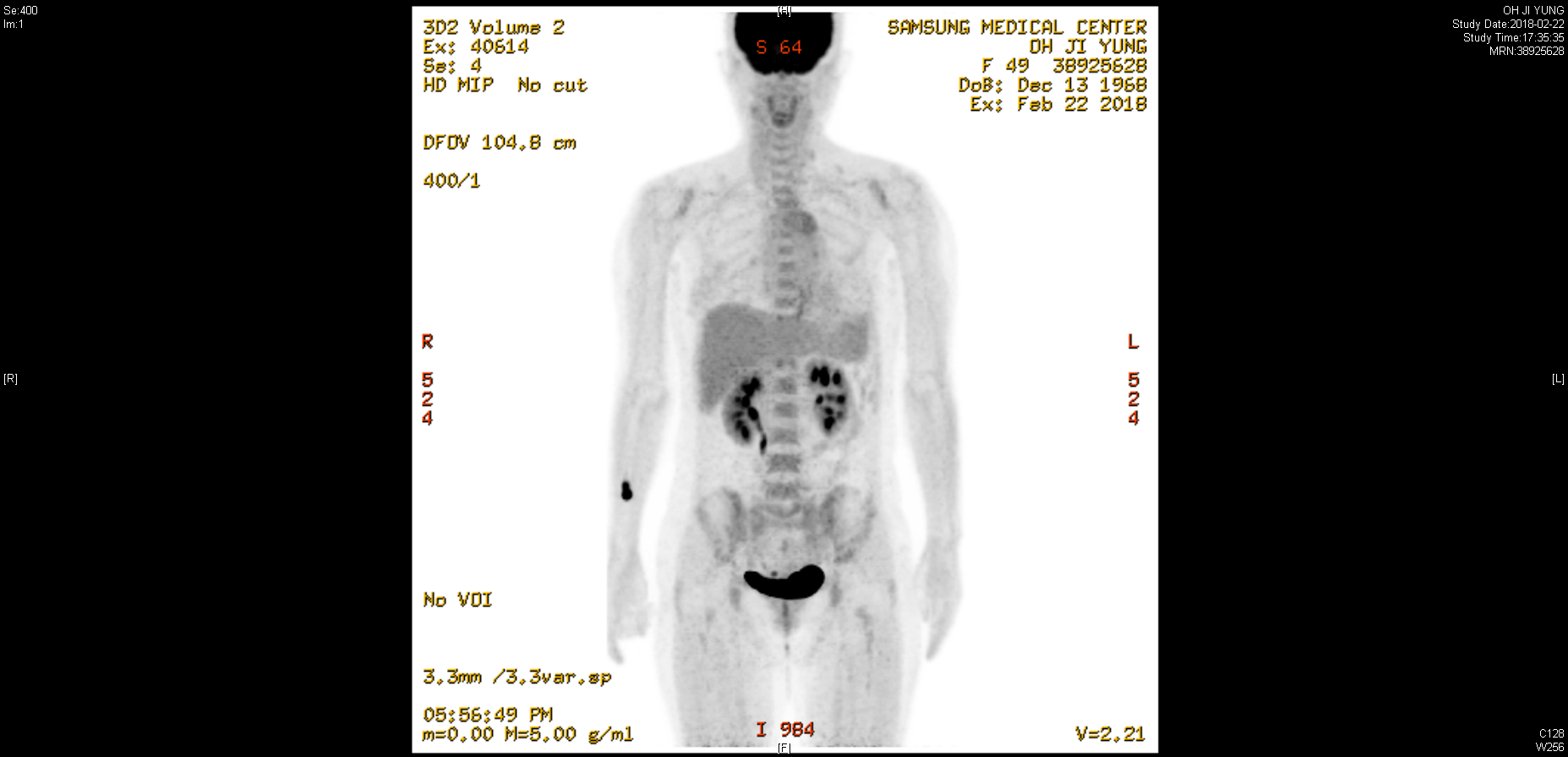

Supplement: Supplementary file 1 — Supplementary Figure. [file 41598_2020_75620_MOESM1_ESM.docx]
